# Sir Ganga Ram Hospital classification of groin and ventral abdominal wall hernias

**DOI:** 10.4103/0972-9941.27720

**Published:** 2006-09

**Authors:** Pradeep K Chowbey, Rajesh Khullar, Magan Mehrotra, Anil Sharma, Vandana Soni, Manish Baijal

**Affiliations:** Minimal Access and Bariatric Surgery Centre, Sir Ganga Ram Hospital, New Delhi - 110 060, India

**Keywords:** Total extraperitoneal repair, SGRH classification, laparoscopic ventral hernia repair

## Abstract

**Background:**

Numerous classifications for groin and ventral hernias have been proposed over the past five to six decades. The old, simple classification of groin hernia in to direct, inguinal and femoral components is no longer adequate to understand the complex pathophysiology and management of these hernias. The most commonly followed classification for ventral hernias divide them into congenital, acquired, incisional and traumatic, which also does not convey any information regarding the predicted level of difficulty.

**Aim:**

All the previous classification systems were based on open hernia repairs and have their own fallacies particularly for uncommon hernias that cannot be classified in these systems. With the advent of laparoscopic/ endoscopic approach, surgical access to the hernia as well as the functional anatomy viewed by the surgeon changed. This change in the surgical approach and functional anatomy opened the doors for newer classifications. The authors have thus proposed a classification system based on the expected level of intraoperative difficulty for endoscopic hernia repair.

**Classification:**

In the proposed classification higher grades signify increasing levels of expected intraoperative difficulty. This functional classification grades groin hernias according to the: a) Pre -operative predictive level of difficulty of endoscopic surgery, and b) Intraoperative factors that lead to a difficult repair. Pre operative factors include multiple or pantaloon hernias, recurrent hernias, irreducible and incarcerated hernias.

Intraoperative factors include reducibility at operation, degree of descent of the hernial sac and previous hernia repairs. Hernial defects greater than 7 cm in diameter are categorized one grade higher.

**Conclusion:**

Though there have been several classification systems for groin or inguinal hernias, none have been described for total classification of all ventral hernias of the abdomen. The system proposed by us includes all abdominal wall hernias and is a final classification that predicts the expected level of difficulty for an endoscopic hernia repair.

## CLASSIFICATION SYSTEMS FOR GROIN HERNIA

Numerous classifications for groin hernia have been proposed over the past five to six decades. The old simple classification of groin hernia into indirect and direct, inguinal and femoral components is no longer adequate to understand the complex pathophysiology and management of these hernias.[1] In the 1950s and 1960s, many surgical classifications for groin hernias were conceived, such as those by Casten,[2] Fruchaud,[3] Harkins[4] and Halverson *et al.*[5] However, they have little applicability in the current surgical practice for hernia. In 1988, Gilbert[6] described a detailed classification based on anatomical and functional defects established intraoperatively and created a registry named ‘Cooperative Hernia Analysis of Types and Surgeries’ (CHATS). In 1991, Nyhus *et al*[7] introduced a classification system based on anatomical criteria with emphasis on the state of the deep ring and posterior wall of the inguinal canal. In 1993, Bendavid[8] proposed the type, staging and dimension system for classification of hernias. All these classification systems based on open hernia repair techniques have their own shortcomings, particularly noninclusion of uncommon hernias that cannot be classified.

Though there have been several classification systems for groin or inguinal hernias, none have been described for total classification of all ventral hernias of the abdomen.[9] The most commonly followed classification system for ventral hernias divides them into (i) congenital hernias - present at birth, which include omphalocele, gastroschisis and umbilical hernia; (ii) acquired - including hernias in the midline, median and paramedian areas, such as Spigelian, epigastric and paraumbilical hernias; (iii) incisional hernias; and (iv) traumatic hernias - following penetrating and blunt trauma.

## SGRH CLASSIFICATION

With the advent of laparoscopic / endoscopic surgery, surgical access to the hernia as well as the functional anatomy viewed by the surgeon changed. The change in the surgical approach and functional anatomy opened the doors for newer classifications. The authors have proposed a classification system based on the expected level of intraoperative difficulty for endoscopic hernia repair. In the proposed classification, higher grades signify increasing levels of expected intraoperative difficulty. A hernial defect >7 cm in diameter is categorized one grade higher.

## CLASSIFICATION OF INGUINAL HERNIA FOR TEP REPAIR

This functional classification grades groin hernias according to the preoperative predictive level of difficulty of endoscopic surgery. For multiple or pantaloon (direct and indirect components) hernias, grading is according to the dominant hernia. Bowel obstruction and strangulation are unsuitable for the total extraperitoneal (TEP) approach. Intraoperatively, the factors considered as predictors of the grade of difficulty include:

- Reducibility
- Degree of descent of the hernial sac
- Previous hernia repair

### Grade I

- Small, direct, reducible hernia
- Swelling appears on coughing / straining and disappears on lying down
- Fingerbreadth size defect in the functional direct floor (Hesselbach's triangle)
- Endoscopically - minimal dissection of sac from fascia transversalis is required

### Grade II

- Small, indirect, incomplete, reducible hernia
- Hernial swelling limited to inguinal canal
- Endoscopically - the sac can be reduced completely and may not require transection or ligation
- Moderate-size direct hernia
- Swelling is present in standing and reduces in the supine position
- Thumb-sized defect in the direct floor
- Endoscopically, the sac needs to be dissected off from the fascia transversalis
- Reducible femoral hernia

### Grade III

- Moderate-size indirect, reducible inguinal hernia
- Hernial swelling (sac) extends beyond superficial ring, up to the neck of scrotum but does not descend to the testis
- Endoscopically - this type of hernia will require transection of sac and ligation of the proximal part of sac
- Large reducible direct hernia
- Involvement of the entire direct floor
- Big bulge on clinical examination over the triangle of Hesselbach
- Endoscopically, creation of space in the midline is difficult. There is anatomical distortion - stretching and lateral displacement of inferior epigastric vessel
- Recurrent groin hernia
- Endoscopically - difficult dissection in region of spermatic cord and the space lateral to it

### Grade IV

- **Large reducible indirect inguino-scrotal hernia**
- Large sac extending up to the testis. The testis cannot be palpated separately from hernia in erect position
- The sac may contain omentum or small bowel, which require manual reduction in supine position
- Endoscopically - the internal ring is enlarged with a wide-mouthed sac. There is difficulty in dissecting sac from cord structures. Medial displacement and stretching of the inferior epigastric vessels may occur. Inadvertent opening of peritoneum may lead to pneumoperitoneum and dissection of sac becomes difficult
- There is higher incidence of postoperative seroma / hematoma because of traction on sac
- The chances of damage to the cord structures are increased

### Grade V

- Large, complete, indirect inguinal hernia, which is only partially reducible or irreducible
- Irreducible femoral hernia
- The sliding component includes the bowel or bladder
- Endoscopically - the sac is bulky. There are adhesions between contents of the sac and sac wall. The sac often needs to be opened and the contents reduced laparoscopically. Injury to the contents (bowel, bladder and omentum) while reducing them is likely

### Notes

- For multiple / pantaloon hernias, ‘difficulty’ grading is according to the dominant hernia.
- Bowel obstruction / strangulation are unsuitable for TEP approach.

## CLASSIFICATION OF VENTRAL HERNIA

### Grade I

- Primary, small, completely reducible ventral hernia
- The location may be umbilical / paraumbilical / epigastric / supravesical / spigelian

### Grade II

- Completely reducible incisional hernia
- The margins of defect should be clearly palpable

### Grade III

- Primary hernia - partially reducible or irreducible
- Contents - omentum only
- Reducible incisional hernia at special operative sites such as Pfannensteil, subcostal incision or extended sternotomy incisions

### Grade IV

- Primary hernia containing bowel, which is partially reducible or irreducible
- More planning in port placement and mesh fixation is required
- Lumbar hernia
- Colon needs to be reflected

### Grade V

- Incisional hernia containing bowel - partially reducible or irreducible
- All margins of defect cannot be clearly felt
- Patients will have symptoms of colic or subacute intestinal obstruction (SAIO) on history and on clinical examination (palpation / auscultation) will reveal presence of bowel in hernial sac

### Grade VI

- Multiple scarred abdomen
- Multiple previous incisions
- Previous hernia repair (recurrent incisional hernia)
- Presenting as acute obstruction

### Note

Patient having colicky intestinal pain or symptoms of SAIO are considered in Grade IV at least. Clinically, on examination bowel loop may give gurgling sensation and reduce partially on palpation. This can be distinguished from omentum on palpation and auscultation.
